# Chinese patent medicines combined with hormone replacement therapy for premature ovarian failure: A Bayesian network meta-analysis

**DOI:** 10.3389/fmed.2022.1043390

**Published:** 2022-11-17

**Authors:** Han-Zhi Zhong, Mao-Ya Li, Xiao-Lan Yin, Cheng-Li Bin, Si-Yun Zhou, Shao-Bin Wei

**Affiliations:** Hospital of Chengdu University of Traditional Chinese Medicine, Chengdu, China

**Keywords:** Chinese patent medicine, hormone replacement therapy (HRT), premature ovarian failure (POF), network meta-analysis (NMA), validity

## Abstract

**Objective:**

The objective of this study was to compare the efficacy differences between Chinese patent medicines combined with hormone replacement therapy (HRT) in the treatment of premature ovarian failure (POF) by the Bayesian network meta-analysis (NMA) method.

**Methods:**

Randomized controlled trials (RCTs) reporting Chinese patent medicine combined with HRT for POF included Medline (*via* PubMed), Embase, Cochrane Library, China National Knowledge Infrastructure Database (CNKI), Wanfang Database (Wanfang), VIP Database (VIP), and China Biology Medicine Database (CBM) from the inception of the databases to July 2022. Two researchers independently screened the articles, extracted data, and evaluated the quality. The literature that met the inclusion criteria was screened out, the quality and risk of bias of the included studies were assessed according to the Cochrane 5.1 manual and RevMan 5.4, and NMA was performed using Stata 15.0 and R software.

**Results:**

Sixty-four RCTs involving 5,675 individuals containing 12 oral Chinese patent medicines combined with HRT were enrolled into the current NMA. The results showed that when compared with patients using only HRT, the total clinical response rate is greater in patients using HRT combined with one of these 12 oral Chinese patent medicines. Among them, Zuogui pills + HRT [odds ratio (OR) = 3.92; 95% credible interval (CrI) = 0.86, 23.84; SUCRA = 73.76%] is most likely to be the best intervention, and the suboptimal intervention is Guishen pills + HRT (OR = 3.22, 95% CrI = 1.16, 9.44, SUCRA = 70.60%).

**Conclusion:**

Chinese patent medicines combined with HRT were more effective than HRT alone in the treatment of POF. Zuogui pills are good at decreasing follicle-stimulating hormone (FSH) and luteinizing hormone (LH) and more effective in the improvement of total clinical response rate; Xuefu Zhuyu capsule is also good at decreasing FSH. Ziheche capsule is an expert in improving estradiol level; Kuntai capsule shows the lowest incidence of adverse reactions. However, the quality of the literature included in this study is relatively low, so it may affect the results of the study. Therefore, higher quality and multi-center trial would be necessary for supporting these results.

**Systematic review registration:**

[www.crd.york.ac.uk/prospero], identifier [CRD42022350587].

## Introduction

Premature ovarian failure (POF) refers to the cessation of ovarian function before the age of 40 and is one of the prominent problems and diseases of female reproductive health. In recent years, the incidence of POF has gradually increased and shows a younger trend. The incidence rate before the age of 40 is about 1%, and the incidence of POF is 1 in 100 women before 40 years of age and 1 in 1,000 women before 30 years of age (1, 2). The main pathogenesis of POF is related to iatrogenic factors, immune factors, and genetic factors. The clinical features of POF are hypoestrogenism or estrogen deficiency, elevated gonadotropin levels, and lack of mature follicles. Estrogen deficiency can cause menopausal symptoms, such as sweating, hot flashes, vaginal and urinary symptoms, and vaginal dryness. However, decreased fertility and even infertility are the top POF-related concerns for women of every reproductive age. In addition, the negative effects of POF include an increased risk of cardiovascular disease, osteoporosis, and sexual dysfunction (3, 4).

In, ESHRE published a guideline for premature ovarian insufficiency, and this review presented the hormone replacement therapy (HRT) options for women with ovarian failure until natural menopause (5). Studies showed that HRT can compensate for estrogen deficiency, resulting in relief of menopausal symptoms (6). Also, it reduces the risk of cardiovascular disease (7, 8) and the impact on bone health in the long run (9–11). Since HRT has been used for a long time and cannot restore ovarian function, some researchers began to find out whether only using traditional Chinese medicine or combined with HRT can enhance the efficacy and gradually restore ovarian function without increasing adverse reactions. A meta-analysis study showed that Kuntai capsule alone had no significant difference compared with HRT in improving clinical efficacy, follicle-stimulating hormone (FSH), luteinizing hormone (LH), and estradiol (E_2_) (12). With the publication of studies on Chinese patent medicine combined with HRT in the treatment of POF, a published systematic review has confirmed the advantages of Chinese patent medicine combined with HRT in the treatment of this disease (13). At present, there are a variety of proprietary Chinese medicines for the treatment of POF, but there are few related clinical studies, and there is a lack of objective evidence-based medical evidence to confirm their safety and effectiveness. At the same time, there is currently a lack of comparison between the efficacy of different Chinese patent medicines. So, it is difficult to evaluate the efficacy and safety of various Chinese patent medicines combined with HRT in the treatment of POF. Therefore, the aim of our study was to rank the effects of various interventions using directly or indirectly available evidence through a Bayesian network meta-analysis (NMA) to gain insight into the strengths and weaknesses of these interventions to provide evidence for clinical treatment.

## Methods

### Registration

This systematic review and NMA are reported in accordance with the Preferred Reporting Items for Systematic Reviews and Meta-Analyses (PRISMA-NMA) statement. The study is registered with the International Prospective Register of Systematic Reviews (registration number CRD42022350587). Ethics approval or patient consent was not required, as all analyses were based on previously published studies.

### Search strategies

Randomized controlled trials (RCTs) reporting Chinese patent medicine combined with HRT for POF included Medline (*via* PubMed), Embase, Cochrane Library, China National Knowledge Infrastructure Database (CNKI), Wanfang Database (Wanfang), VIP Database (VIP), and China Biology Medicine Database (CBM) from the inception of the databases to July 2022. The following Medical Subject Headings [MeSH] and keywords incorporating Boolean operators were applied: “premature ovarian insufficiency,” “primary ovarian insufficiency,” “POF,” “Chinese herbal,” “Traditional Chinese Medicine,” “Chinese and Western Medicine,” “capsule,” “grain,” “Oral liquid,” “pill,” “Dan,” “Gao.” We also implemented a recursive manual search to search full-text studies from obtained tracking bibliographies or similar systematic reviews to check for potentially qualified studies that we missed first.

### Inclusion criteria

The **inclusion criteria** were constructed around the PICOS standard:

1)participants: The included subjects are all patients with POF [at least 4 months of oligomenorrhea or amenorrhea; two random measurements (> 4 weeks) of FSH > 40 IU/L] (14);

2)interventions: Chinese patent medicine combined with HRT;

3)comparison: HRT alone;

4)outcomes: main outcomes: total clinical response rate [(recovery number of cases + efficiency number of cases)/total number of cases]; secondary outcomes: serum FSH, LH, estradiol (E_2_) levels, and adverse reactions;

5)study design: randomized clinical trial (RCT).

### Exclusion criteria

**The exclusion criteria are as follows:**

1)observation group or control group combined with other treatment methods;

2)literature that cannot extract complete outcome indicators or the full text cannot be obtained;

3)self-control studies, non-randomized controlled trial, experimental studies, experience summary, reviews, and case reports;

4)excluded studies in which the full text could not be obtained, the same data were repeatedly published, the use of HRT courses were unreasonable, and there were no diagnostic criteria for the disease.

### Literature screening and data extraction

Selecting the studies will be accomplished by importing them into Endnote 20 (version 20.1.0, Clarivate Analytics) to manage and remove duplicate entries. Having independently screened the literature to determine whether it meets inclusion criteria, two researchers then read the abstracts and full texts to determine whether they meet the criteria. Data extraction content includes publication, patient information, intervention and control measures, treatment course, and outcome indicators.

### Quality evaluation

The quality of assessment was according to Cochrane Handbook for Systematic Reviews of Interventions (15), including the following seven domains. Each of these options was evaluated as high, low, or unclear. For selection bias, it was defined as low risk of bias if the study described the method of sequence generation and allocation concealment, otherwise it was considered high risk of bias. For performance bias, the study was considered low risk of bias if it describes the method used to blind subjects, otherwise it was considered high risk of bias. For measurement bias, studies were considered low risk of bias if they described all methods of blinding outcome assessors, otherwise high risk of bias. For follow-up bias, studies were considered low risk of bias if the study described completeness of outcome data for each primary outcome (including loss to follow-up, data excluded from analysis), and high risk of bias otherwise. For reporting bias, studies were considered low risk of bias if they described how systematic reviewers examined selective outcome reporting that may have occurred, and high risk of bias otherwise. For other biases, studies were considered to be at high risk of bias if the study design was imprecise, or reporting was significantly inconsistent with previous studies. Seven items were rated as “unclear risk” when the study did not mention relevant items. Any disagreement will be resolved through discussion with the superior researcher.

### Certainty of the evidence

The grading of recommendations assessment, development, and evaluation (GRADE) approach (16–18) for NMA was used to rate the certainty of the evidence of NMA estimates. Comparisons were initially rated as high-quality evidence and were downgraded accordingly, based on study limitations, imprecision, inconsistency, indirectness, and publication bias. We downgraded the study quality by one level in the study limitation, including concerns about selection bias, performance bias, detection bias, attrition bias, reporting bias, or other bias; as for imprecision item, if the sample size is insufficient or an imprecise estimate of the wide confidence interval is produced in this comparison, we will downgrade. For inconsistencies, we downgraded it by one level if study heterogeneity was found in the comparison, particularly local inconsistencies between direct and indirect evidence. If heterogeneity is observed according to four areas, namely demographic disparities, interventions, outcome measurement, and indirect comparisons, indirect projects will be downgraded. For publication bias item, we judge by asymmetric funnel diagrams. After the above assessment, the quality of evidence will be classified into one of four levels, including high, moderate, low, and very low quality. Two investigators rated the certainty of consulting with a third party.

### Statistical analyses

The direct pairwise meta-analysis and bias evaluated were performed with RevMan 5.4. Variables with continuous and categorical effects were measured using the odds ratio (OR) and mean difference (MD). OR and MD were calculated using 95% credible interval [CrI]. In terms of heterogeneity, *I*^2^ represents the statistical value of 25, 50, and 75% of mild, moderate, and high heterogeneity, respectively, and was used to measure the presence or absence of substantial heterogeneity (19).

Network transitivity was considered a crucially important assumption in NMA, and its evaluation will further directly influence our analysis (20). Therefore, to ensure that multiple treatment comparisons were sufficiently similar, we estimated transmissibility by comparing clinical and methodological characteristics (e.g., patients and experimental design) across all included studies (15). The STATA/SE version 15.0 (StataCorp, College Station, TX) was used to drawn network plots and comparison-adjusted funnel plot. We employed R Version 5.0 (Mac OS X 12_5_1) with the GeMTC package to conduct NMA. The parameters in GeMTC were set as follows: initial value, 2.5; number of simulation iterations, 50,000; number of annealing times, 20,000; thinning factor, 1; and number of chains, 4. The convergence of iterations can be monitored in terms of potential scale reduction factors (PSRFs). To rank the effects of the intervention, we used the cumulative ranking probability curve (SUCRA, surface under the cumulative ranking area) and found that higher SUCRA values indicate greater efficacy (21). We did not implement the hypothesis of consistency because of non-close loops. Finally, identifying evidence of small-sample effects in networks by plotting comparative corrected funnel plots.

## Results

### Literature retrieval process and results

**FIGURE 1:**
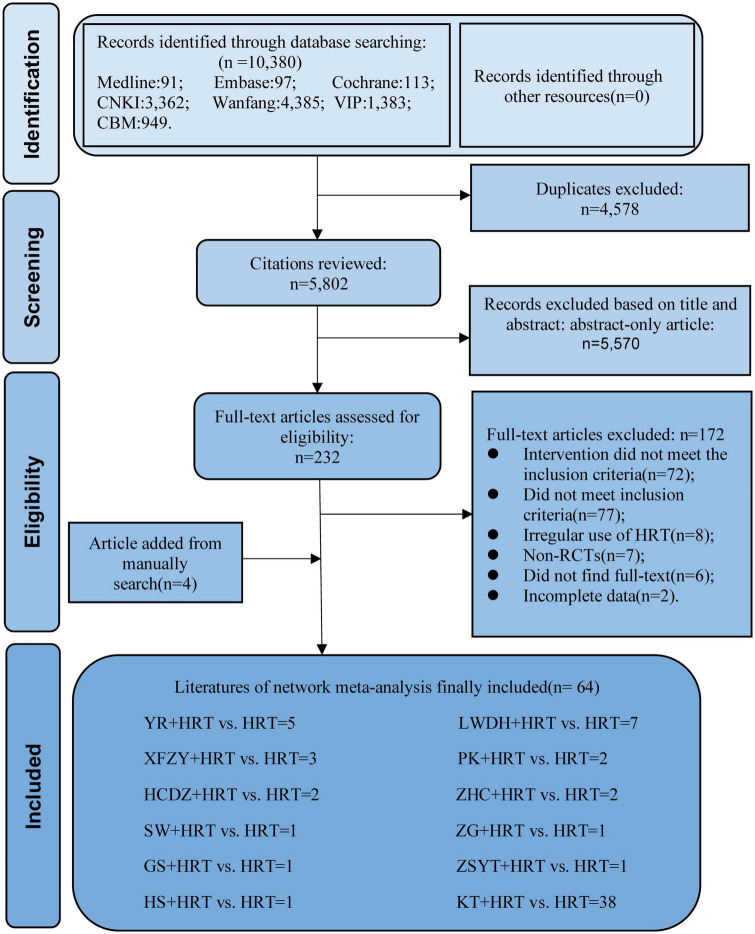
Literature review flowchart.

A total of 10,380 literature from seven databases were included in this study. First, we have removed duplicate 4,578 studies and, by reading the title and abstract, removed incompatible 5,570 studies. Second, by reading the full text, 127 incompatible studies were removed. The reasons for exclusion are as follows: (1) intervention did not meet the inclusion criteria (*n* = 72); (2) did not meet inclusion criteria (*n* = 77); (3) irregular use of HRT (*n* = 8); (4) non-RCTs (*n* = 7); (5) did not find full text (*n* = 6); (6) incomplete data (*n* = 2). Finally, 64 articles (22–85) were included for research. The selection process is illustrated in Figure 1.

### Quality of the included studies

All 64 RCTs were mentioned randomization, of which 30 were random number table method, one was simple random method, and one was the envelope lottery method, rated as low risk. The rest were only described as “random” and rated as unclear risk; in terms of allocation concealment, blinding of participants or doctors, and blinding of outcome evaluator, all articles were not described and rated as unclear risk; all articles with complete data and no selective reporting were rated as unclear risk, unable to judge other sources of bias, rated as unclear risk. The assessment results of the risk of bias test are shown in Supplementary Figure 1. The GRADE level of evidence for the primary outcome was rated very low to low quality, shown in Supplementary Table 1.

### Network meta-analysis

**FIGURE 2:**
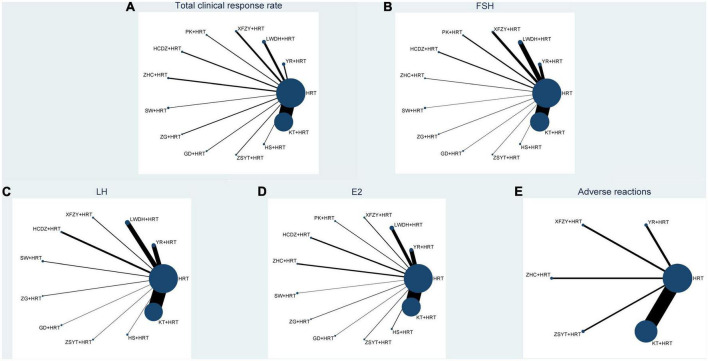
Network plot of all the trials based on the outcomes. P.S: **(A)** total clinical response rate; **(B)** FSH; **(C)** LH; **(D)** E2; **(E)** adverse reactions; HRT, hormone replacement therapy; YR, Fuke Yangrong Capsule; LWDH, Liuwei Dihuang Pills; XFZY, Xuefu Zhuyu Capsule; PK, Peikun pills; HCDZ, Heche Dazao pills; ZHC, Ziheche Capsule; SW, Siwu Mixture; ZG, Zuogui pills; GS, Guishen pills; ZSYT, Zishen Yutai pills; HS, Huanshao Capsule; KT, Kuntai Capsule.

Figure 2 shows all outcome measures, with all included Chinese patent medicines combined with HRT being compared at least once, while there is a lack of closed loop between various types of Chinese patent medicines combined with HRT.

### Primary outcome

#### Total clinical response rate

A total of 52 studies involving 4,702 patents reported the total clinical response rate. Among them, GS + HRT, HCDZ + HRT, SW + HRT, KT + HRT, XFZY + HRT, YR + HRT, and LWDH + HRT compared with HRT have statistical significance (see Table 1). ZG + HRT [odds ratio (OR) = 3.92; 95% credible interval (CrI) 0.86, 23.84; SUCRA = 73.76%] had the highest rate of impact on POF patients in terms of the other 11 Chinese patent medicines combined with HRT. Next, the second was GS + HRT (OR = 3.22, 95% CrI = 1.16, 9.44, SUCRA = 70.60%) and HCDZ + HRT (OR = 2.95, 95% CrI = 1.35, 6.51, SUCRA = 68.41%) was third. The comparison-correction funnel plot showed that not all studies were symmetrically distributed around the X = 0 line, and two studies were located outside the funnel chart, which provides evidence for small-sample effects in the study network (see in Supplementary Figure 2). None of the five outcome indicators in this study exhibited a closed loop, so there is no necessary to do a consistency test.

**TABLE 1:** Relative effect sizes of efficacy at post-treatment according to network meta-analysis.

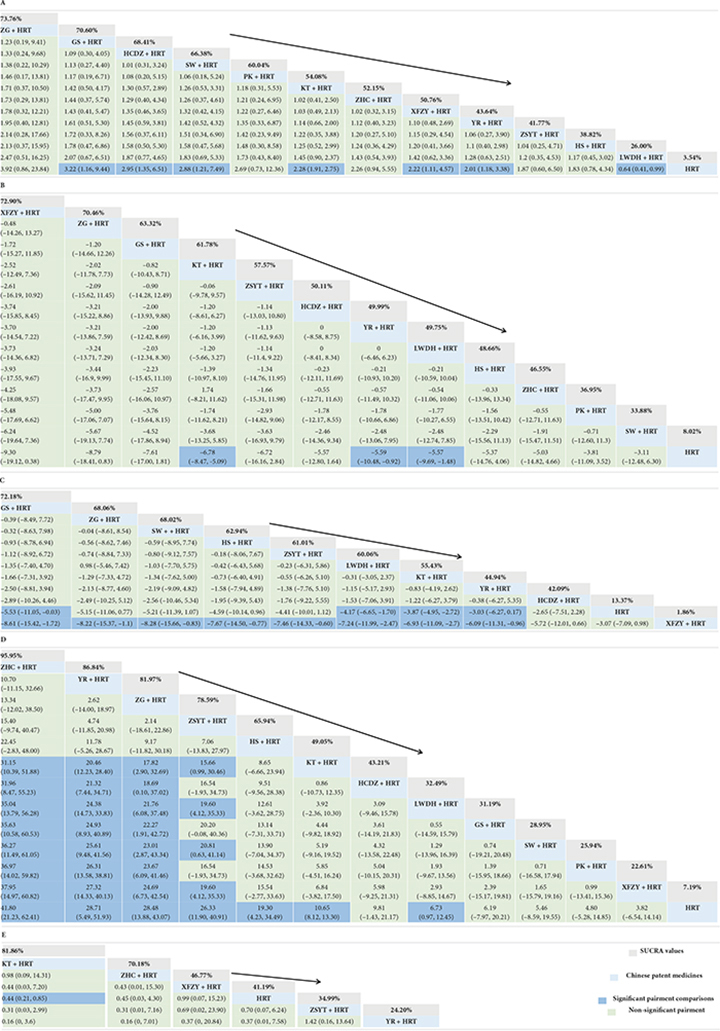

HRT, hormone replacement therapy; YR, Fuke Yangrong Capsule; LWDH, Liuwei Dihuang Pills; XFZY, Xuefu Zhuyu Capsule; PK, Peikun pills; HCDZ, Heche Dazao pills; ZHC, Ziheche Capsule; SW, Siwu Mixture; ZG, Zuogui pills; GS, Guishen pills; ZSYT, Zishen Yutai pills; HS, Huanshao Capsule; KT, Kuntai Capsule. SUCRA, surface under the cumulative ranking. **(A)** Total clinical response rate; **(B)** FSH; **(C)** LH; **(D)** E2; **(E)** adverse reactions; Highest probability of being the most efficient treatment (With high SUCRA values) and Lowest probability of being the most efficient treatment (With low SUCRA values). The bold part is the SUCRA value, which has been color-coded and annotated and the other bold part is the intervention name.

### Secondary outcome

A total of 59 studies involving 5,415 patents reported FSH. Only KT + HRT (MD = –6.78, 95% CrI = –8.47, –5.09), YR + HRT (MD = –5.59, 95% CrI = –10.48, –0.92), and LWDH + HRT (MD = –5.57, 95% CrI = –9.69, –1.48) had a significant benefit compared with HRT, and other Chinese patent medicines combined with HRT had no statistical differences. According to the SUCRA value, XFZY + HRT was ranked first (SUCRA = 72.90%) (see Table 1).

There are 51 studies involving 4,629 patents reported LH. Only GS + HRT (MD = –5.53, 95% CrI = –11.05, –0.03), LWDH + HRT (MD = –4.17, 95% CrI = –6.65, –1.7), KT + HRT (MD = –3.87, 95% CrI = –4.95, –2.72), and YR + HRT (MD = –3.03, 95% CrI = –6.27, –0.17) had a significant benefit compared with HRT, and other Chinese patent medicines combined with HRT had no statistical differences. According to the SUCRA value, GS + HRT was ranked first (SUCRA = 72.18%) (see Table 1).

A total of 58 studies involving 5,315 patents reported E_2_. ZHC + HRT (MD = 41.8; 95% CrI = 21.23, 62.41; SUCRA = 95.95%) had the highest rate of impact on POF patients in terms of the other 11 Chinese patent medicines combined with HRT. Next, the second was YR + HRT (MD = 28.71, 95% CrI = 5.49, 51.93, SUCRA = 86.84%), and ZG + HRT (MD = 28.48, 95% CrI = 13.88, 43.07, SUCRA = 81.97%) was third (see Table 1).

A total of 13 studies involving 1,168 patents reported the adverse reactions. Only KT + HRT had a significant benefit compared with HRT. KT + HRT (OR = 0.44, 95% CrI = 0.21, 0.85, SUCRA = 81.86%) shows the best, and ZHC + HRT (OR = 0.45, 95% CrI = 0.03, 4.3, SUCRA = 70.18%) was as follows. The comparison-correction funnel plot of all secondary outcome is shown in Supplementary Figure 2.

## Discussion

Our study has adopted a Bayesian NMA which involves 64 RCTs to evaluate the effectiveness of 12 Chinese patent medicines combined with HRT in POF patients. In terms of primary outcome, ZG + HRT appears to be the most promising way to help patients with POF improve their clinically integrated outcomes.

In recent years, with the development of social economy, women’s increasing mental stress and poor living habits have led to the gradual rejuvenation of patients with POF, which is a devastating diagnosis for women of childbearing age, because it indicates a decline in fertility (1, 86). As a first-line treatment widely used in patients with POF, HRT can improve the clinical symptoms caused by estrogen deficiency in patients, but it is not insisted on by patients because it cannot restore ovarian function and the treatment time is too long (recommended until natural menopause) (5). At present, in addition to hormone therapy, there are still complementary therapies, such as inositol, vitamin D, and traditional Chinese medicine. Inositol has been found to be a natural endogenous compound, which includes D-Chiro-Inositol (DCI) and Myo-inositol (MI), of which MI is the second messenger of insulin and FSH, which can improve insulin resistance (87), correct the FSH/LH ratio, and promote ovulation (88); DCI can improve the quality of oocytes and blastocysts, which has a potential role in improving fertility (89). Similarly, the fact that vitamin D receptors are present in women’s central and peripheral reproductive organs, tissues, and cells suggests that vitamin D plays a key role in fertility. Vitamin D supplementation promotes oocyte development, improves embryo quality, and increases endometrial tolerance (90); in addition, inositol can reduce fasting insulin, blood total cholesterol, blood triglycerol levels, thereby reducing diabetes, dyslipidemia, and cardiovascular disease risk, vitamin D supplementation can also reduce the risk of osteoporosis, and no adverse reactions have been shown, which is undoubtedly a significant advantage for perimenopausal women (91).

In addition, for patients of reproductive age with POF, how to protect fertility at a young age is a key concern. Vitrification of oocytes is an effective technique for fertility protection that allows women to preserve gametes for future fertility in advance. One prospective study found no statistically significant difference in pregnancy and clinical pregnancy rates per cycle between vitrified oocytes and sibling fresh oocytes in closed systems (92). Another prospective study found that open and closed vitrification protocols were equally effective for sibling oocyte cycles when performing blastocyst embryo transfers (93). Thus, vitrification of oocytes offers women the possibility of delaying fertility until the end of treatment or finding a suitable time for fertility. However, the psychological support given during the treatment of infertile patients is also a point that cannot be ignored. POF can cause depression and anxiety disorders in most women. In terms of fertility, studies have found that due to gender differences, infertile couples, although they are seriously affected by infertility diagnosis, in terms of behavior, relationship, social, emotional, and cognitive aspects, regardless of the way they are conceived, women are more likely than men to “seek social support” (94, 95). Therefore, understanding the sources and changes of psychological stress in female patients and providing patients with specific psychotherapy can benefit them in terms of interventions and outcomes of infertility treatment.

Proprietary Chinese medicine has always been a hot topic in Chinese medical research and is widely used in the clinical frontline, especially by gynecologists and fertility center doctors in general hospitals. Based on this purpose, we conducted a study of proprietary Chinese medicine combined with HRT in the treatment of POF, trying to obtain relatively objective evidence, hoping to give full play to the advantages of the three combination of the two, gradually restore ovarian function, to shorten the treatment time, and look for the best efficacy of proprietary Chinese medicine combined with HRT to provide an evidence base. The essence of POF is considered to be the depletion of follicles, and experimental studies have found that Zuogui pills may improve the therapeutic effect of chemotherapy-induced POF by inhibiting the pathway of mitochondrial-dependent apoptosis, laying an experimental foundation for Zuogui pills as a reasonable treatment choice for POF (96). A meta-analysis study also shows that ZG + HRT is more therapeutic and safer than HRT alone (97). Combined with the findings of this study, in the primary outcome, total clinical response rate, ZG + HRT (OR = 3.92; 95% CrI = 0.86, 23.84) is the best in all treatments. Therefore, we can cautiously think that ZG + HRT seems to be the most effective proprietary Chinese medicine in terms of improving total clinical response rate.

This study showed that XFZY + HRT (SUCRA = 72.90%) worked best at reducing FSH levels, but it has no statistical differences. Some scholars found that the ovarian artery peak systolic velocity in POF patients was negatively correlated with FSH (98). Modern pharmacological studies have found that the extract of Honghai–Taoren drug pair can promote blood circulation by affecting hemodynamics, plasma coagulation, and platelet aggregation (99, 100). XFTZ Capsule is composed of a variety of traditional Chinese medicines for promoting blood circulation and removing blood stasis, including Honghai and Taoren. So we infer that XFZY Capsule may reduce FSH levels by improving hemodynamics and improving ovarian blood supply, but this inference lacks experimental research. For the outcome of E_2_, ZHC + HRT (MD = 41.8; 95% CrI = 21.23, 62.41; SUCRA = 95.95%) was best in all treatments. The composition of Ziheche Capsule is Ziheche. Modern pharmacological studies have found that Ziheche contains a large number of hormones, including gonadotropin, corticotropin-releasing hormone, thyrotropin, prolactin, a variety of LHs, and erythropoietin, which can directly stimulate ovarian tissue and promote endometrial hyperplasia (101). Therefore, we can cautiously conclude that ZHC + HRT has the best effect in improving the E_2_ level. However, outcome measures for many interventions were not statistically significant, so more long-term and high-quality, large-sample, multi-center RCTs are needed in future to further confirm this.

### Innovation and limitations of this study

In this study, for the first time, a NMA was used to classify and analyze the relevant efficacy indicators of proprietary Chinese patient medicines combined with HRT in the treatment of POF. Giving full play to the advantages of proprietary Chinese patient medicines, it provides evidence-based evidence for reasonable and targeted drugs in clinical practice. In addition, this study searched seven databases and finally included 64 RCTs involving 5,675 POF patients, with a large sample size and many sources of evidence.

At the same time, this study has certain limitations: (1) There are differences in the number of studies included in different interventions. For example, there are 42 studies involving Kuntai capsule, and only one study involving Zuogui pills, Guishen pills, Zishen Yutai pills, Huanshao capsule, and Siwu mixture. The difference in number may affect the results of this study make an impact; (2) in this study, 12 proprietary Chinese patient medicines were indirectly compared with HRT, and there was no RCTs that directly compared the efficacy of proprietary Chinese patient medicines, and the evidence network graphs of the five outcome indicators did not form a closed loop, which affected the credibility and stability of the results to a certain extent. (3) In terms of literature quality evaluation, all studies were single-center, Chinese literature, and most of the random methods used the random number table method, and none of them mentioned the allocation concealment, the blinding method of subjects and outcome evaluators, and other sources of bias could not be judged. Therefore, we look forward to conducting more RCTs with large samples, multicenter, and high methodological quality in future to provide more robust and reliable evidence support for clinical drug use.

## Data availability statement

The original contributions presented in this study are included in the article/Supplementary material, further inquiries can be directed to the corresponding author.

## Author contributions

H-ZZ served as principal author, had full access to all the data in the study, took responsibility for the accuracy of the data analysis and the integrity of the data, and contributed to the draft of the manuscript. C-LB and M-YL contributed to the conception and design. X-LY, C-LB, and S-YZ contributed to data acquisition and interpretation. X-LY, M-YL, and S-BW contributed to revising of the article and final approval. All authors contributed to the article and approved the submitted version.
