# Supplementary material for: Chinese patent medicines combined with hormone replacement therapy for premature ovarian failure: A Bayesian network meta-analysis
Source: Front Med (Lausanne). 2022 Nov 17;9:1043390. doi: 10.3389/fmed.2022.1043390 (PMC9712806; doi:10.3389/fmed.2022.1043390)
Supplement: Supplementary file 1 [file Data_Sheet_1.pdf]

Appendix : Search strategy

Randomized controlled trials(RCTs) reporting Chinese patent medicine combined with HRT for POF included by Medline(PubMed), Embase, Cochrane Library, China National Knowledge Infrastructure Database(CNKI), Wanfang Database(Wanfang), VIP Database(VIP), and China Biology Medicine Database(CBM) from the inception of the databases to July 2022.

The search terms are as following: (premature ovarian insufficiency OR primary ovarian insufficiency OR premature ovarian failure) AND (Chinese herbal OR Traditional Chinese Medicine OR Chinese and Western Medicine OR capsule OR grain OR Oral liquid OR pill OR Dan OR Gao). The search strategies for PubMed are shown in **Table 1**.We will review and investigate the titles and abstracts of all literature to eliminate repetitive or irrelevant articles.

Table 1 | Search strategy for PubMed

**Number**	**Search Terms**
1	premature ovarian insufficiency
2	primary ovarian insufficiency
3	premature ovarian failure
4	or 1-3
5	Chinese herbal
6	Traditional Chinese Medicine
7	Chinese and Western Medicine
8	capsule
9	grain
10	Oral liquid
11	pill
12	Dan
13	Gao
14	or 4-13
15	4 and 14
