# Supplementary figures and images for: Chinese patent medicines combined with hormone replacement therapy for premature ovarian failure: A Bayesian network meta-analysis
Source: Front Med (Lausanne). 2022 Nov 17;9:1043390. doi: 10.3389/fmed.2022.1043390 (PMC9712806; doi:10.3389/fmed.2022.1043390)

**Supplementary Figure 1 |** Results of the bias risk assessment of the included studies

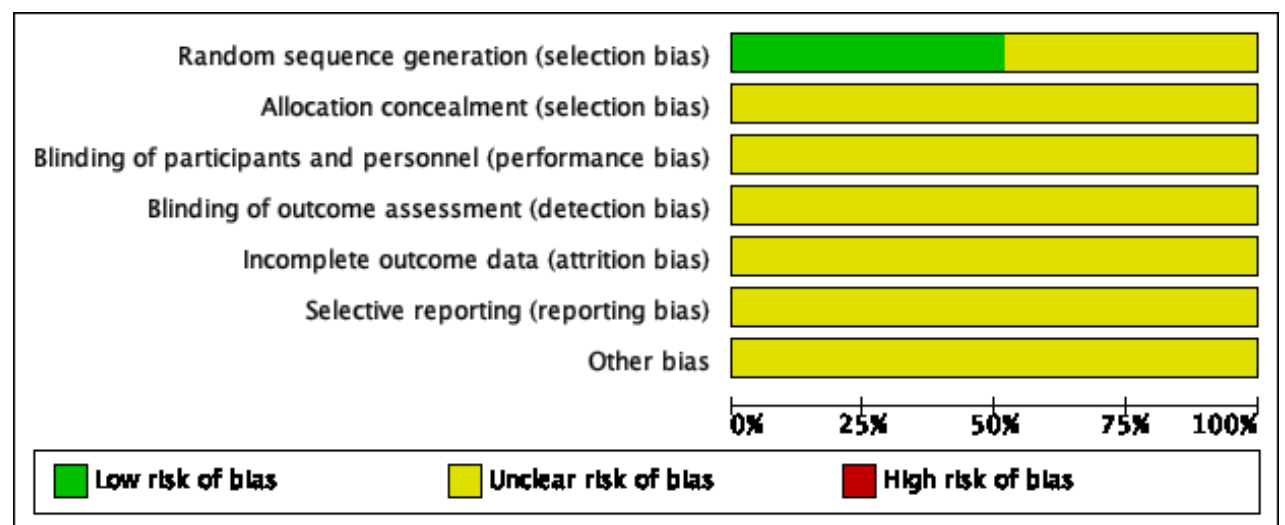

Supplement: Supplementary file 2 [file Image_1.pdf]
