# Supplementary material for: Chinese patent medicines combined with hormone replacement therapy for premature ovarian failure: A Bayesian network meta-analysis
Source: Front Med (Lausanne). 2022 Nov 17;9:1043390. doi: 10.3389/fmed.2022.1043390 (PMC9712806; doi:10.3389/fmed.2022.1043390)
Supplement: Supplementary file 3 [file Image_2.pdf]

Supplementary Figure 2 | funnel chart

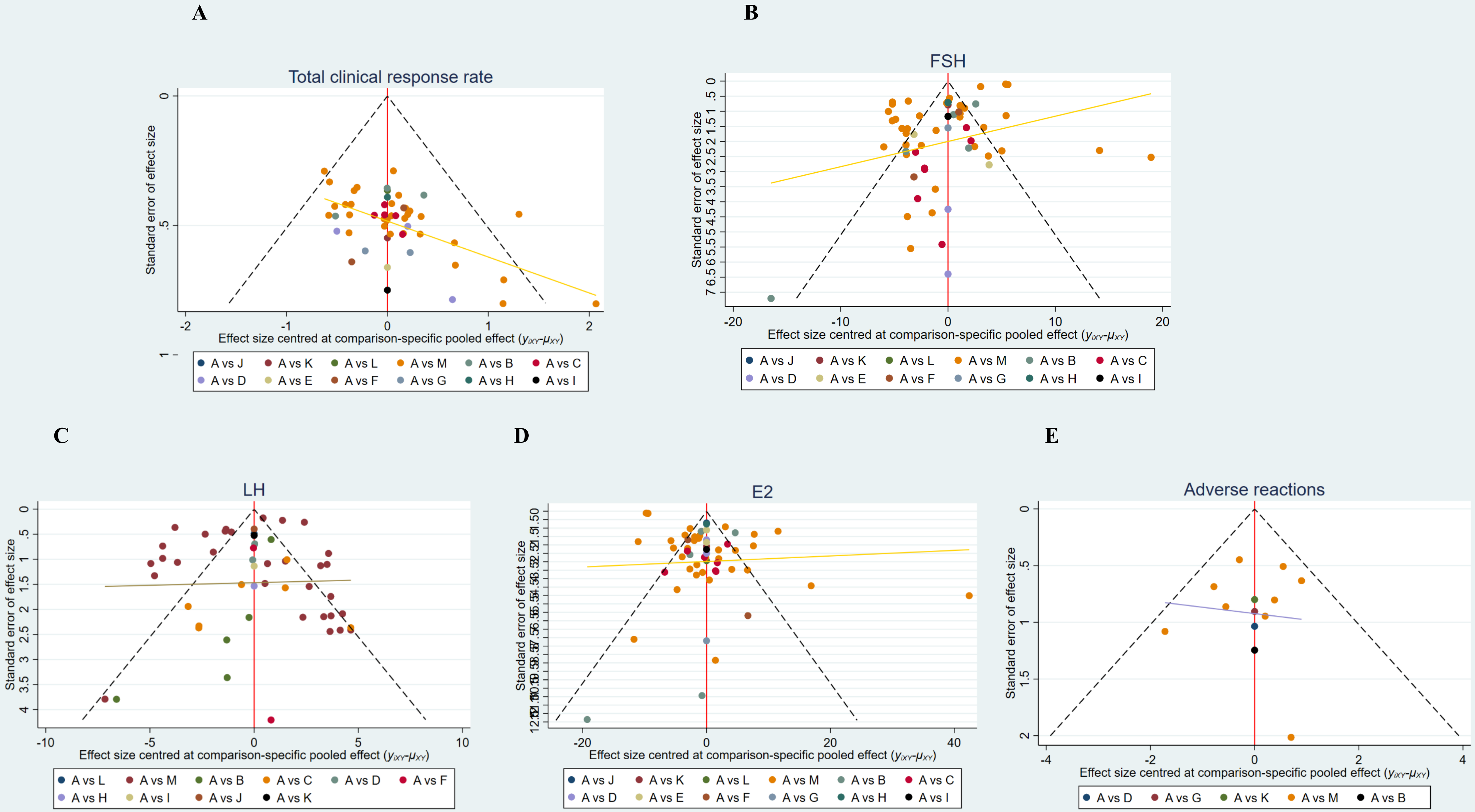

P.S: A, hormone replacement therapy(HRT); B, Fuke Yangrong Capsule(YR) + HRT; C, Liuwei Dihuang Pills(LWDH) + HRT; D, Xuefu Zhuyu Capsule(XFZY) +HRT; E, Peikun pills(PK) + HRT; F, Heche Dazao pills(HCDZ) +HRT; G, Ziheche Capsule(ZHC) + HRT; H, Siwu Mixture(SW) + HRT; I, Zuogui pills(ZG) + HRT; J, Guishen pills(GS) + HRT; K, Zishen Yutai pills(ZSYT) + HRT; L, Huanshao Capsule(HS) +HRT; M, Kuntai Capsule(KT) +HRT; **(A)** total clinical response rate; **(B)** FSH; **(C)** LH; **(D)** E2; **(E)** adverse reactions.
