# Supplementary material for: Chinese patent medicines combined with hormone replacement therapy for premature ovarian failure: A Bayesian network meta-analysis
Source: Front Med (Lausanne). 2022 Nov 17;9:1043390. doi: 10.3389/fmed.2022.1043390 (PMC9712806; doi:10.3389/fmed.2022.1043390)
Supplement: Supplementary file 4 [file Table_1.pdf]

Quality of Evidence for Outcomes

YR + HRT vs HRT for POF

Patient or population: patients with POF

Settings:

Intervention: YR+HRT vs HRT

**Outcomes**	**Illustrative comparative risks* (95% CI)**		**Relative effect (95% CI)**	**No of Participants (studies)**	**Quality of the evidence (GRADE)**	**Comments**
	Assumed risk	Corresponding risk				
	**Control**	**YR + HRT vs HRT**				
**Total clinical response rate**	**Study population**		**OR 1.99** (1.28 to 3.1)	325 (3 studies)	⊕⊕⊖⊖ **low**[1,2]	
	**373 per 1000**	**542 per 1000** (432 to 648)				
	**Medium risk population**					
	**400 per 1000**	**570 per 1000** (460 to 674)				
**FSH**		The mean FSH in the intervention groups was **3.28 lower** (4.42 to 2.14 lower)		447 (5 studies)	⊕⊕⊖⊖ **low**[1,3]	
**LH**		The mean LH in the intervention groups was **1.62 lower** (2.71 to 0.53 lower)		447 (5 studies)	⊕⊕⊖⊖ **low**[1,3]	
**E2**		The mean E2 in the intervention groups was **33.33 higher**		447 (5 studies)	⊕⊕⊖⊖ **low**[1,3]	

		(31.71 to 34.94 higher)			
**Adverse reactions**	**Study population**		**OR 2.05** (0.18 to 23.59)	80 (1 study)	⊕⊖⊖⊖ **very low**[1,2,4]
	**25 per 1000**	**50 per 1000** (5 to 377)			
	**Medium risk population**				
	**25 per 1000**	**50 per 1000** (5 to 377)			

*The basis for the **assumed risk** (e.g. the median control group risk across studies) is provided in footnotes. The **corresponding risk** (and its 95% confidence interval) is based on the assumed risk in the comparison group and the **relative effect** of the intervention (and its 95% CI).

CI: Confidence interval; **OR:** Odds ratio;

GRADE Working Group grades of evidence

High quality: Further research is very unlikely to change our confidence in the estimate of effect.

Moderate quality: Further research is likely to have an important impact on our confidence in the estimate of effect and may change the estimate.

Low quality: Further research is very likely to have an important impact on our confidence in the estimate of effect and is likely to change the estimate.

Very low quality: We are very uncertain about the estimate.

[1] The blinding and distribution concealment methods was no metion in the literature, and there were methodological flaws.

[2] The number of cases included in the literature was low.

[3] The funnel chart indicated publication bias.

[4] Only one document was included.

LWDH + HRT vs HRT for POF

Patient or population: patients with POF

Settings:

Intervention: LWDH + HRT

Outcomes	Illustrative comparative risks* (95% CI)		Relative effect (95% CI)	No of Participants (studies)	Quality of the evidence (GRADE)	Comments
	Assumed risk	Corresponding risk				
	**Control**	**LWDH + HRT vs HRT**				
**Total clinical response rate**	**Study population**		**OR 1.56** (1.04 to 2.35)	409 (5 studies)	⊕⊕⊖⊖ **low**[1,2]	
	**325 per 1000**	**429 per 1000** (334 to 531)				
	**Medium risk population**					
	**350 per 1000**	**457 per 1000** (359 to 559)				
**FSH**		The mean FSH in the intervention groups was **5.29 lower** (7.34 to 3.23 lower)		481 (7 studies)	⊕⊕⊖⊖ **low**[1,2]	
**LH**		The mean LH in the intervention groups was **3.75 lower** (4.96 to 2.54 lower)		590 (7 studies)	⊕⊕⊖⊖ **low**[1,2]	
**E2**		The mean E2 in the intervention groups was **14.12 higher** (12.18 to 16.07 higher)		590 (7 studies)	⊕⊕⊖⊖ **low**[1,2]	

*The basis for the **assumed risk** (e.g. the median control group risk across studies) is provided in footnotes. The **corresponding risk** (and its 95% confidence interval) is based on the assumed risk in the comparison group and the **relative effect** of the intervention (and its 95% CI).

CI: Confidence interval; **OR:** Odds ratio;

GRADE Working Group grades of evidence

High quality: Further research is very unlikely to change our confidence in the estimate of effect.

Moderate quality: Further research is likely to have an important impact on our confidence in the estimate of effect and may change the estimate.

Low quality: Further research is very likely to have an important impact on our confidence in the estimate of effect and is likely to change the estimate.

Very low quality: We are very uncertain about the estimate.

[1] The blinding and distribution concealment methods was no metion in the literature, and there were methodological flaws.

[2] The funnel chart indicated publication bias.

XFZY + HRT vs HRT for POF

Patient or population: patients with HRT

Settings:

Intervention: XFZY + HRT

Outcomes	Illustrative comparative risks* (95% CI)		Relative effect (95% CI)	No of Participants (studies)	Quality of the evidence (GRADE)	Comments
	Assumed risk	Corresponding risk				
	**Control**	**XFZY + HRT vs HRT**				
**Total clinical response rate**	**Study population**		**OR 2.17** (1.15 to 4.1)	158 (3 studies)	⊕⊖⊖⊖ **very low**[1,2,3]	
	**342 per 1000**	**530 per 1000** (374 to 681)				
	**Medium risk population**					

	**324 per 1000**	**510 per 1000**<br>(355 to 663)			
**FSH**		The mean FSH in the intervention groups was<br>**9.3 lower**<br>(16.24 to 2.36 lower)		98<br>(2 studies)	⊕⊝⊝⊝<br>**very low**[1,2,3]
**LH**		The mean LH in the intervention groups was<br>**3.07 higher**<br>(1.95 to 4.19 higher)		98<br>(2 studies)	⊕⊝⊝⊝<br>**very low**[1,2,3]
**E2**		The mean E2 in the intervention groups was<br>**3.8 higher**<br>(1.04 to 6.56 higher)		98<br>(2 studies)	⊕⊝⊝⊝<br>**very low**[1,2,3]
**Adverse reactions**	**Study population**		**OR 1**<br>(0.13 to 7.6)	60<br>(1 study)	⊕⊝⊝⊝<br>**very low**[1,2,3]
	**67 per 1000**	**67 per 1000**<br>(9 to 353)			
	**Medium risk population**				
	**67 per 1000**	**67 per 1000**<br>(9 to 353)			

*The basis for the **assumed risk** (e.g. the median control group risk across studies) is provided in footnotes. The **corresponding risk** (and its 95% confidence interval) is based on the assumed risk in the comparison group and the **relative effect** of the intervention (and its 95% CI).

CI: Confidence interval; **OR:** Odds ratio;

GRADE Working Group grades of evidence

High quality: Further research is very unlikely to change our confidence in the estimate of effect.

Moderate quality: Further research is likely to have an important impact on our confidence in the estimate of effect and may change the estimate.

Low quality: Further research is very likely to have an important impact on our confidence in the estimate of effect and is likely to change the estimate.

Very low quality: We are very uncertain about the estimate.

[1] The blinding and distribution concealment methods was no metion in the literature, and there were methodological flaws.

[2] The number of cases included in the literature was low.

[3] The funnel chart indicated publication bias.

PK + HRT vs HRT for POF

Patient or population: patients with POF

Settings:

Intervention: PK + HRT

Outcomes	Illustrative comparative risks* (95% CI)		Relative effect (95% CI)	No of Participants (studies)	Quality of the evidence (GRADE)	Comments
	Assumed risk	Corresponding risk				
	**Control**	**PK + HRT vs HRT**				
**Total clinical response rate**	**Study population**		**OR 2.62** (0.72 to 9.61)	65 (1 study)	⊕⊖⊖⊖ **very low**[1,2,3]	
	**125 per 1000**	**272 per 1000** (93 to 579)				
	**Medium risk population**					
	**125 per 1000**	**272 per 1000** (93 to 579)				
**FSH**		The mean FSH in the intervention groups was **4.99 lower**		89 (2 studies)	⊕⊖⊖⊖ **very low**[1,2,4]	

		(7.9 to 2.07 lower)			
**E2**		The mean E2 in the intervention groups was **4.8 higher** (2.92 to 6.68 higher)		89 (2 studies)	⊕⊝⊝⊝ **very low**[1,2,4]

*The basis for the **assumed risk** (e.g. the median control group risk across studies) is provided in footnotes. The **corresponding risk** (and its 95% confidence interval) is based on the assumed risk in the comparison group and the **relative effect** of the intervention (and its 95% CI).

CI: Confidence interval; **OR:** Odds ratio;

GRADE Working Group grades of evidence

High quality: Further research is very unlikely to change our confidence in the estimate of effect.

Moderate quality: Further research is likely to have an important impact on our confidence in the estimate of effect and may change the estimate.

Low quality: Further research is very likely to have an important impact on our confidence in the estimate of effect and is likely to change the estimate.

Very low quality: We are very uncertain about the estimate.

[1] The blinding and distribution concealment methods was no metion in the literature, and there were methodological flaws.

[2] The number of cases included in the literature was low.

[3] Only one document was included.

[4] The funnel chart indicated publication bias.

HCDZ + HRT vs HRT for POF

Patient or population: patients with POF

Settings:

Intervention: HCDZ + HRT

Outcomes	Illustrative comparative risks* (95% CI)		Relative effect (95% CI)	No of Participants (studies)	Quality of the evidence (GRADE)	Comments
	Assumed risk	Corresponding risk				
	**Control**	**HCDZ + HRT vs HRT**				
**Total clinical response rate**	**Study population**		**OR 2.91** (1.44 to 5.86)	135 (2 studies)	⊕⊕⊖⊖ **low**[1,2]	
	**358 per 1000**	**619 per 1000** (445 to 766)				
	**Medium risk population**					
	**356 per 1000**	**617 per 1000** (443 to 764)				
**FSH**		The mean FSH in the intervention groups was **4.24 lower** (6.15 to 2.33 lower)		135 (2 studies)	⊕⊖⊖⊖ **very low**[1,2,3]	
**LH**		The mean LH in the intervention groups was **2.83 lower** (4.33 to 1.34 lower)		135 (2 studies)	⊕⊖⊖⊖ **very low**[1,2,3]	
**E2**		The mean E2 in the intervention groups was **6.95 higher** (3.74 to 10.16 higher)		135 (2 studies)	⊕⊖⊖⊖ **very low**[1,2,3]	
**Adverse reactions**	See comment	See comment	Not estimable	135 (2 studies)	⊕⊕⊖⊖ **low**[1,3]	

*The basis for the **assumed risk** (e.g. the median control group risk across studies) is provided in footnotes. The **corresponding risk** (and its 95% confidence interval) is based on the assumed risk in the comparison group and the **relative effect** of the intervention (and its 95% CI).

CI: Confidence interval; **OR:** Odds ratio;

GRADE Working Group grades of evidence

High quality: Further research is very unlikely to change our confidence in the estimate of effect.

Moderate quality: Further research is likely to have an important impact on our confidence in the estimate of effect and may change the estimate.

Low quality: Further research is very likely to have an important impact on our confidence in the estimate of effect and is likely to change the estimate.

Very low quality: We are very uncertain about the estimate.

[1] The blinding and distribution concealment methods was no metion in the literature, and there were methodological flaws.

[2] The funnel chart indicated publication bias.

[3] The number of cases included in the literature was low.

ZHC + HRT vs HRT for POF

Patient or population: patients with POF

Settings:

Intervention: ZHC + HRT

Outcomes	Illustrative comparative risks* (95% CI)		Relative effect (95% CI)	No of Participants (studies)	Quality of the evidence (GRADE)	Comments
	Assumed risk	Corresponding risk				
	**Control**	**ZHC + HRT vs HRT**				
**Total clinical response rate**	**Study population**		**OR 2.2** (0.96 to 5.05)	135 (2 studies)	⊕⊖⊖⊖ **very low**[1,2,3]	
	**162 per 1000**	**298 per 1000** (157 to 494)				
	**Medium risk population**					
	**162 per 1000**	**298 per 1000** (157 to 494)				

**FSH**		The mean FSH in the intervention groups was **5.07 lower** (8.11 to 2.03 lower)		65 (1 study)	⊕⊖⊖⊖ **very low**[1,2,4]
**E2**		The mean E2 in the intervention groups was **41.79 higher** (26.73 to 56.85 higher)		65 (1 study)	⊕⊖⊖⊖ **very low**[1,2,4]
**Adverse reactions**	**Study population**		**OR 0.48** (0.08 to 2.84)	65 (1 study)	⊕⊖⊖⊖ **very low**[1,2,4]
	**121 per 1000**	**62 per 1000** (11 to 281)			
	**Medium risk population**				
	**121 per 1000**	**62 per 1000** (11 to 281)			

*The basis for the **assumed risk** (e.g. the median control group risk across studies) is provided in footnotes. The **corresponding risk** (and its 95% confidence interval) is based on the assumed risk in the comparison group and the **relative effect** of the intervention (and its 95% CI).

CI: Confidence interval; **OR:** Odds ratio;

GRADE Working Group grades of evidence

High quality: Further research is very unlikely to change our confidence in the estimate of effect.

Moderate quality: Further research is likely to have an important impact on our confidence in the estimate of effect and may change the estimate.

Low quality: Further research is very likely to have an important impact on our confidence in the estimate of effect and is likely to change the estimate.

Very low quality: We are very uncertain about the estimate.

[1] The blinding and distribution concealment methods was no metion in the literature, and there were methodological flaws.

[2] The number of cases included in the literature was low.

[3] The funnel chart indicated publication bias.

[4] Only one document was included.

SW + HRT vs HRT for POF

Patient or population: patients with POF

Settings:

Intervention: SW + HRT

**Outcomes**	**Illustrative comparative risks* (95% CI)**		**Relative effect (95% CI)**	**No of Participants (studies)**	**Quality of the evidence (GRADE)**	**Comments**
	Assumed risk	Corresponding risk				
	**Control**	**SW + HRT vs HRT**				
**Total clinical response rate**	**Study population**		**OR 2.86** (1.33 to 6.16)	164 (1 study)	⊕⊕⊖⊖ **low**[1,2]	
	**671 per 1000**	**854 per 1000** (731 to 926)				
	**Medium risk population**					
	**671 per 1000**	**854 per 1000** (731 to 926)				
**FSH**		The mean FSH in the intervention groups was **3.1 lower** (4.5 to 1.7 lower)		164 (1 study)	⊕⊖⊖⊖ **very low**[1,2,3]	
**LH**		The mean LH in the intervention groups was **5.2 lower** (8.21 to 2.19 lower)		164 (1 study)	⊕⊖⊖⊖ **very low**[1,2,3]	

**E2**		The mean E2 in the intervention groups was **5.5 higher** (4.01 to 6.99 higher)		164 (1 study)	⊕⊖⊖⊖ **very low**[1,2,3]

*The basis for the **assumed risk** (e.g. the median control group risk across studies) is provided in footnotes. The **corresponding risk** (and its 95% confidence interval) is based on the assumed risk in the comparison group and the **relative effect** of the intervention (and its 95% CI).

CI: Confidence interval; **OR:** Odds ratio;

GRADE Working Group grades of evidence

High quality: Further research is very unlikely to change our confidence in the estimate of effect.

Moderate quality: Further research is likely to have an important impact on our confidence in the estimate of effect and may change the estimate.

Low quality: Further research is very likely to have an important impact on our confidence in the estimate of effect and is likely to change the estimate.

Very low quality: We are very uncertain about the estimate.

[1] The blinding and distribution concealment methods was no metion in the literature, and there was methodological flaw.

[2] Only one document was included.

[3] The number of cases included in the literature was low.

ZG + HRT vs HRT for POF

Patient or population: patients with POF

Settings:

Intervention: ZG + HRT

Outcomes	Illustrative comparative risks* (95% CI)		Relative effect (95% CI)	No of Participants (studies)	Quality of the evidence (GRADE)	Comments
	Assumed risk	Corresponding risk				
	**Control**	**ZG + HRT vs HRT**				

**Total clinical response rat**	**Study population**		**OR 3.45** (0.79 to 15.01)	50 (1 study)	⊕⊝⊝⊝ **very low**[1,2,3]
	**120 per 1000**	**320 per 1000** (97 to 672)			
	**Medium risk population**				
	**120 per 1000**	**320 per 1000** (97 to 672)			
**FSH**		The mean FSH in the intervention groups was **8.81 lower** (11.1 to 6.52 lower)		50 (1 study)	⊕⊝⊝⊝ **very low**[1,2,3]
**LH**		The mean LH in the intervention groups was **5.15 lower** (7.38 to 2.92 lower)		50 (1 study)	⊕⊝⊝⊝ **very low**[1,2,3]
**E2**		The mean E2 in the intervention groups was **28.47 higher** (24.02 to 32.92 higher)		50 (1 study)	⊕⊝⊝⊝ **very low**[1,2,3]

*The basis for the **assumed risk** (e.g. the median control group risk across studies) is provided in footnotes. The **corresponding risk** (and its 95% confidence interval) is based on the assumed risk in the comparison group and the **relative effect** of the intervention (and its 95% CI).

CI: Confidence interval; **OR:** Odds ratio;

GRADE Working Group grades of evidence

High quality: Further research is very unlikely to change our confidence in the estimate of effect.

Moderate quality: Further research is likely to have an important impact on our confidence in the estimate of effect and may change the estimate.

Low quality: Further research is very likely to have an important impact on our confidence in the estimate of effect and is likely to change the estimate.

Very low quality: We are very uncertain about the estimate.

[1] The blinding and distribution concealment methods was no metion in the literature, and there were methodological flaws.

[2] The number of cases included in the literature was low.

[3] Only one document was included.

GS + HRT vs HRT for POF

Patient or population: patients with POF

Settings:

Intervention: GS + HRT

Outcomes	Illustrative comparative risks* (95% CI)		Relative effect (95% CI)	No of Participants (studies)	Quality of the evidence (GRADE)	Comments
	Assumed risk	Corresponding risk				
	Control	GS + HRT vs HRT				
**Total clinical response rate**	**Study population**		**OR 3.16** (1.25 to 7.96)	82 (1 study)	⊕⊖⊖⊖ **very low**[1,2,3]	
	**268 per 1000**	**536 per 1000** (314 to 745)				
	**Medium risk population**					
	**268 per 1000**	**536 per 1000** (314 to 745)				
**FSH**		The mean FSH in the intervention groups was **7.59 lower** (8.94 to 6.24 lower)		82 (1 study)	⊕⊖⊖⊖ **very low**[1,2,3]	
**LH**		The mean LH in the intervention groups was **5.54 lower**		82 (1 study)	⊕⊖⊖⊖ **very low**[1,2,3]	

	(6.32 to 4.76 lower)				
**E2**	The mean E2 in the intervention groups was **6.2 higher** (4.82 to 7.58 higher)		82 (1 study)	⊕⊖⊖⊖ **very low**[1,2,3]	

*The basis for the **assumed risk** (e.g. the median control group risk across studies) is provided in footnotes. The **corresponding risk** (and its 95% confidence interval) is based on the assumed risk in the comparison group and the **relative effect** of the intervention (and its 95% CI).

CI: Confidence interval; **OR:** Odds ratio;

GRADE Working Group grades of evidence

High quality: Further research is very unlikely to change our confidence in the estimate of effect.

Moderate quality: Further research is likely to have an important impact on our confidence in the estimate of effect and may change the estimate.

Low quality: Further research is very likely to have an important impact on our confidence in the estimate of effect and is likely to change the estimate.

Very low quality: We are very uncertain about the estimate.

[1] The blinding and distribution concealment methods was no metion in the literature, and there were methodological flaws.

[2] The number of cases included in the literature was low.

[3] Only one document was included.

ZSYT + HRT vs HRT for POF

Patient or population: patients with POF

Settings:

Intervention: ZSYT + HRT

Outcomes	Illustrative comparative risks* (95% CI)	Relative effect	No of Participants	Quality of the evidence	Comments

	Assumed risk<br>**Control**	Corresponding risk<br>**ZSYT + HRT vs HRT**	**(95% CI)**	**(studies)**	**(GRADE)**
**Total clinical response rate**	**Study population**		**OR 1.8**<br>(0.61 to 5.26)	78<br>(1 study)	⊕⊖⊖⊖<br>**very low**[1,2,3]
	**179 per 1000**	**282 per 1000**<br>(117 to 534)			
	**Medium risk population**				
	**180 per 1000**	**283 per 1000**<br>(118 to 536)			
**FSH**		The mean FSH in the intervention groups was<br>**6.71 lower**<br>(8.26 to 5.16 lower)		78<br>(1 study)	⊕⊖⊖⊖<br>**very low**[1,2,3]
**LH**		The mean LH in the intervention groups was<br>**4.41 lower**<br>(5.44 to 3.38 lower)		78<br>(1 study)	⊕⊖⊖⊖<br>**very low**[1,2,3]
**E2**		The mean E2 in the intervention groups was<br>**26.39 higher**<br>(22.31 to 30.47 higher)		78<br>(1 study)	⊕⊖⊖⊖<br>**very low**[1,2,3]
**Adverse reactions**	**Study population**		**OR 1.37**<br>(0.29 to 6.58)	78<br>(1 study)	⊕⊖⊖⊖<br>**very low**[1,2,3]
	**77 per 1000**	**103 per 1000**<br>(24 to 354)			
	**Medium risk population**				
	**77 per 1000**	**103 per 1000**<br>(24 to 354)			

*The basis for the **assumed risk** (e.g. the median control group risk across studies) is provided in footnotes. The **corresponding risk** (and its 95% confidence interval) is based on the assumed risk in the comparison group and the **relative effect** of the intervention (and its 95% CI).

CI: Confidence interval; **OR:** Odds ratio;

GRADE Working Group grades of evidence

High quality: Further research is very unlikely to change our confidence in the estimate of effect.

Moderate quality: Further research is likely to have an important impact on our confidence in the estimate of effect and may change the estimate.

Low quality: Further research is very likely to have an important impact on our confidence in the estimate of effect and is likely to change the estimate.

Very low quality: We are very uncertain about the estimate.

[1] The blinding and distribution concealment methods was no metion in the literature, and there were methodological flaws.

[2] The number of cases included in the literature was low.

[3] Only one document was included.

HS + HRT vs HRT for POF

Patient or population: patients with POF

Settings:

Intervention: HS + HRT

**Outcomes**	**Illustrative comparative risks* (95% CI)**		**Relative effect (95% CI)**	**No of Participants (studies)**	**Quality of the evidence (GRADE)**	**Comments**
	Assumed risk **Control**	Corresponding risk **HS + HRT vs HRT**				
**Total clinical response rate**	**Study population**		**OR 1.8** (0.88 to 3.69)	132 (1 study)	⊕⊕⊖⊖ **low**[1,2]	
	**303 per 1000**	**439 per 1000** (277 to 616)				

	**Medium risk population**				
	**303 per 1000**	**439 per 1000**<br>(277 to 616)			
**FSH**		The mean FSH in the intervention groups was<br>**5.38 lower**<br>(6.71 to 4.05 lower)		132<br>(1 study)	⊕⊖⊖⊖<br>**very low**[1,2,3]
**LH**		The mean LH in the intervention groups was<br>**4.61 lower**<br>(5.57 to 3.65 lower)		132<br>(1 study)	⊕⊖⊖⊖<br>**very low**[1,2,3]
**E2**		The mean E2 in the intervention groups was<br>**19.35 higher**<br>(13.6 to 25.1 higher)		132<br>(1 study)	⊕⊖⊖⊖<br>**very low**[1,2,3]
**Adverse reactions**	See comment	See comment	Not estimable	132<br>(1 study)	⊕⊖⊖⊖<br>**very low**[1,2,3]

*The basis for the **assumed risk** (e.g. the median control group risk across studies) is provided in footnotes. The **corresponding risk** (and its 95% confidence interval) is based on the assumed risk in the comparison group and the **relative effect** of the intervention (and its 95% CI).

CI: Confidence interval; **OR:** Odds ratio;

GRADE Working Group grades of evidence

High quality: Further research is very unlikely to change our confidence in the estimate of effect.

Moderate quality: Further research is likely to have an important impact on our confidence in the estimate of effect and may change the estimate.

Low quality: Further research is very likely to have an important impact on our confidence in the estimate of effect and is likely to change the estimate.

Very low quality: We are very uncertain about the estimate.

[1] The blinding and distribution concealment methods was no metion in the literature, and there were methodological flaws.

[2] Only one document was included.

[3] The number of cases included in the literature was low.

KT + HRT vs HRT for POF

Patient or population: patients with POF

Settings:

Intervention: KT + HRT

Outcomes	Illustrative comparative risks* (95% CI)		Relative effect (95% CI)	No of Participants (studies)	Quality of the evidence (GRADE)	Comments
	Assumed risk	Corresponding risk				
	**Control**	**KT + HRT vs HRT**				
**Total clinical response rate**	**Study population**		**OR 2.3** (1.96 to 2.69)	2831 (30 studies)	⊕⊕⊝⊝ **low**[1,2]	
	**468 per 1000**	**669 per 1000** (633 to 703)				
	**Medium risk population**					
	**414 per 1000**	**619 per 1000** (581 to 655)				
**FSH**		The mean FSH in the intervention groups was **2.37 lower** (2.5 to 2.24 lower)		3407 (34 studies)	⊕⊕⊝⊝ **low**[1,2]	
**LH**		The mean LH in the intervention groups was **3.84 lower** (4.03 to 3.65 lower)		2775 (29 studies)	⊕⊕⊝⊝ **low**[1,2]	

**E2**		The mean E2 in the intervention groups was<br>**1.26 higher**<br>(1.09 to 1.42 higher)		3307<br>(33 studies)	⊕⊕⊝⊝<br>**low**[1,2]
**Adverse reactions**	**Study population**		**OR 0.46**<br>(0.3 to 0.72)	886<br>(9 studies)	⊕⊕⊝⊝<br>**low**[1,2]
	**152 per 1000**	**76 per 1000**<br>(51 to 114)			
	**Medium risk population**				
	**189 per 1000**	**97 per 1000**<br>(65 to 144)			

*The basis for the **assumed risk** (e.g. the median control group risk across studies) is provided in footnotes. The **corresponding risk** (and its 95% confidence interval) is based on the assumed risk in the comparison group and the **relative effect** of the intervention (and its 95% CI).

CI: Confidence interval; **OR:** Odds ratio;

GRADE Working Group grades of evidence

High quality: Further research is very unlikely to change our confidence in the estimate of effect.

Moderate quality: Further research is likely to have an important impact on our confidence in the estimate of effect and may change the estimate.

Low quality: Further research is very likely to have an important impact on our confidence in the estimate of effect and is likely to change the estimate.

Very low quality: We are very uncertain about the estimate.

[1] The blinding and distribution concealment methods was no metion in the literature, and there were methodological flaws.

[2] The funnel chart indicated publication bias.

P.S: HRT, hormone replacement therapy; YR, Fuke Yangrong Capsule; LWDH, Liuwei Dihuang Pills; XFZY, Xuefu Zhuyu Capsule; PK, Peikun pills; HCDZ, Heche Dazao pills; ZHC, Ziheche Capsule; SW, Siwu Mixture; ZG, Zuogui pills; GS, Guishen pills; ZSYT, Zishen Yutai pills; HS, Huanshao Capsule; KT, Kuntai Capsule.
