# Supplementary material for: Chinese patent medicines combined with hormone replacement therapy for premature ovarian failure: A Bayesian network meta-analysis
Source: Front Med (Lausanne). 2022 Nov 17;9:1043390. doi: 10.3389/fmed.2022.1043390 (PMC9712806; doi:10.3389/fmed.2022.1043390)
Supplement: Supplementary file 5 [file Table_2.pdf]

Supplementary Table 2 | Basic features of the include studies.

Treatment measures	Control measures	Publication	Number of included cases		Age(year, x̄±s)		Course of disease (months, x̄±s)		Duration	Outcome
			Trial group	Control group	Trial group	Control group	Trial group	Control group		
YR+HRT	HRT	Chinese and Foreign Medical Research	65	65	32.10 ± 3.90	31.80 ± 3.70	39.20 ± 6.00	39.00 ± 5.80	3 months	①②③④⑤
		Medical Journal of Chinese People's Health	40	40	27.27 ± 2.40	29.08 ± 2.47	15.60 ± 2.40	15.10 ± 2.20	3 months	②③④⑤
		Chinese Journal of Woman and Child Health Research	60	60	NR	NR	NR	NR	3 months	①②③④
		Contemporary Medical Symposium	36	36	33.64 ± 2.17	33.28 ± 2.49	35.07 ± 2.92	35.45 ± 2.86	3 months	①②③④
		CJTCMP	22	23	33.84 ± 6.66	33.95 ± 6.70	NR	NR	3 months	②③④
LWDH+HRT	HRT	Chinese Archives of Traditional Chinese Medicine	30	32	32.4 ± 3.83	32.3 ± 3.64	22.6 ± 12.1	2.23 ± 1.29	3 months	①②③④
		Shenzhen Journal of Integrated Traditional Chinese and Western Medicine	40	40	32.70 ± 4.30	34.24 ± 25.20	12.40 ± 4.10	13.17 ± 3.55	9 months	①②③④
		Bengbu Med Coll	40	41	34.10 ± 3.80	35.20 ± 3.50	9.40 ± 2.90	9.20 ± 4.10	6 months	②③④
		Healthmust-Readmagazine	40	40	31.85 ± 1.15	32.69 ± 1.28	10.21 ± 1.79	10.64 ± 1.81	3 months	①②③④
		Journal of Clinical Medical	39	39	32.78 ± 4.36	NR	12.45 ± 4.12	NR	9 months	①②③④
		Hebei Medicine	61	48	NR	NR	NR	NR	6 months	①②③④
		World Chinese Medicine	50	50	NR	NR	NR	NR	3 months	②③④
XFZY+HRT	HRT	Chinese Journal of Trauma and Disability Medicine	15	15	34.20 ± 3.40	33.50 ± 3.20	25.50 ± 4.20	25.60 ± 4.30	3 months	①②③④
		Journal of Practical Traditional Chinese Medicine	34	34	33.10 ± 2.90	32.80 ± 2.70	2.68 ± 0.54	2.71 ± 0.56	3 months	①②③④

Treatment measures	Control measures	Publication	Number of included cases		Age(year, $\bar{x}$±s)		Course of disease (months, $\bar{x}$±s)		Duration	Outcome
			Trial group	Control group	Trial group	Control group	Trial group	Control group		
XFZY+HRT	HRT	Sichuan Medical Journal	30	30	NR	NR	NR	NR	3 months	①⑤
PK+HRT	HRT	Journal of New Chinese Medicine	33	32	NR	NR	NR	NR	5 months	①②④
		Cardiovascular Disease Journal of integrated traditional Chinese and Western Medicine	12	12	NR	NR	NR	NR	1 months	②④
HCDZ+HRT	HRT	Anhui University Chinese Medicine	21	20	32.10 ± 3.40	33.90 ± 4.20	15.48 ± 5.76	14.39 ± 5.36	3 months	①②③④⑤
		Clinical Research and Practice	47	47	30.23 ± 4.93	29.57 ± 4.46	14.60 ± 4.10	15.80 ± 4.30	3 months	①②③④⑤
ZHC+HRT	HRT	Guide of China Medicine	32	33	33.10 ± 5.58	32.90 ± 5.13	NR	NR	4 months	①②④⑤
		Zhejiang Journal of TCM	35	35	32.10 ± 5.46	32.65 ± 5.11	12.76 ± 7.56	13.00 ± 6.44	4 months	①
SW+HRT	HRT	Journal of New Chinese Medicine	82	82	34.78 ± 5.32	35.06 ± 5.49	28.90 ± 4.60	26.70 ± 5.00	6 months	①②③④
ZG+HRT	HRT	Modern Journal of Integrated Traditional Chinese and Western Medicine	25	25	35.50 ± 2.10	45.30 ± 5.20	13.80 ± 2.80	13.60 ± 2.90	3 months	①②③④
GS+HRT	HRT	Journal of Practical Traditional Chinese Medicine	41	41	29.87 ± 5.76	29.78 ± 5.81	13.10 ± 3.90	12.90 ± 4.10	3 months	①②③④
ZSYT+HRT	HRT	Journal of Hunan University of Chinese Medicine	39	39	28.20 ± 3.20	28.50 ± 3.00	27.00 ± 4.00	28.00 ± 5.00	6 months	①②③④⑤
HS+HRT	HRT	Drugs & Clinic	66	66	35.50 ± 4.00	34.70 ± 3.80	27.50 ± 9.50	26.20 ± 8.80	4 months	①②③④⑤
KT+HRT	HRT	Journal of New Chinese Medicine	41	41	32.54 ± 4.13	31.62 ± 4.04	20.40 ± 5.60	21.30 ± 6.10	3 months	①⑤
		Henan Medical Research	63	63	34.54 ± 2.80	34.76 ± 2.74	15.60 ±7.00	15.20 ± 7.10	6 months	①②③④⑤
		Chinese Journal of Practical Medicine.	51	51	31.75 ± 3.81	32.07 ± 3.95	NR	NR	3 months	①②③④⑤

Treatment measures	Control measures	Publication	Number of included cases		Age(year, $\bar{x}$±s)		Course of disease (months, $\bar{x}$±s)		Duration	Outcome
			Trial group	Control group	Trial group	Control group	Trial group	Control group		
KT+HRT	HRT	Chinese Journal of Practical Medicine.	51	51	31.75 ± 3.81	32.07 ± 3.95	NR	NR	3 months	①②③④⑤
		Henan Medical Research	50	50	33.69 ± 2.51	33.72 ± 2.46	12.50 ±3.50	12.70 ± 3.30	3 months	②③④
		Maternal and Child Health Care of China	30	30	30.71 ± 5.03	30.44 ± 5.11	16.80 ± 2.10	17.60 ± 1.90	6 months	①②③④
		Clinical Research and Practice	100	100	33.68 ± 4.11	33.71 ± 4.15	12.40 ± 3.60	12.90 ± 3.20	3 months	①②③④
		Chinese Journal of Ethnomedicine and Ethnopharmacy	46	46	32.80 ± 6.90	33.60 ± 7.60	37.00 ± 6.00	35.00 ± 7.00	3 months	①②③④
		Chinese Journal of General Practice	45	45	36.20 ± 3.90	36.20 ± 4.10	31.00 ± 5.00	32.00 ± 6.00	3 months	②④
		Shenzhen Journal of Integrated Traditional Chinese and Western Medicine	30	30	33.20 ± 3.50	33.40 ± 3.20	NR	NR	3 months	①②③④⑤
		Electronic Journal of Practical Gynecologic Endocrinology	50	50	35.00 ± 3.30	34.60 ± 3.50	31.30 ± 8.80	31.50 ± 8.90	3 months	②③④
		China Pharmaceuticals	53	52	NR	NR	NR	NR	6 months	①②③④⑤
		Henan Medical Research	39	39	34.17 ± 2.39	34.67 ± 2.48	NR	NR	3 months	①
		China Continuing Medical Education	45	45	33.79 ± 2.27	34.15 ± 2.39	25.10 ± 5.80	24.50 ± 6.10	3 months	①②③④
		Hubei Journal of TCM	40	40	34.20 ± 2.80	34.10 ± 3.00	31.00 ± 11.00	33.00 ± 9.00	3 months	①②③④
		Medicine in Practice	39	39	32.47 ± 3.52	33.14 ± 3.46	23.60 ± 7.30	25.30 ± 6.50	3 weeks	①②③④
		Super Baby	20	20	33.10 ± 4.50	32.80 ± 4.30	NR	NR	2 months	①
		Woman's Health Research	56	56	33.10 ± 3.45	32.67 ± 3.09	NR	NR	3 months	①②④

Treatment measures	Control measures	Publication	Number of included cases		Age(year, $\bar{x}$±s)		Course of disease (months, $\bar{x}$±s)		Duration	Outcome
			Trial group	Control group	Trial group	Control group	Trial group	Control group		
		Medical Journal of Chinese People's Health	47	42	30.98 ± 2.74	31.09 ± 2.83	15.05 ± 2.98	14.36 ± 3.16	3 months	①②③④
		Modern Journal of Integrated Traditional Chinese and Western Medicine	34	34	33.30 ± 3.70	35.30 ± 4.10	15.40 ± 9.20	16.10 ± 8.60	3 months	①②③④⑤
		Journal of Heze Medical College	54	54	NR	NR	NR	NR	50 days	②③④
		Medical Equipment	45	45	34.80 ± 3.80	34.20 ± 3.70	31.00 ± 7.00	28.00 ± 5.00	6 months	①②④
		Henan Medical Research	90	90	32. 97 ± 2.14	33. 41 ± 2.09	27.60 ± 9.10	21.60 ± 5.20	3 months	②③④
		Harbin Medical Journal	47	47	35.12 ± 2.77	35.03 ± 2.98	32.00 ± 8.70	32.20 ± 8.80	3 months	①②③④
		Journal of Practical Gynecologic Endocrinology	73	73	36.30 ± 2.10	36.00 ± 2.50	11.80 ± 2.60	12.40 ± 2.40	3 months	①②③④
KT+HRT	HRT	Journal of Practical Traditional Chinese Medicine	44	44	30.95 ± 3.53	30.26 ± 3.12	25.70 ± 2.40	25.20 ± 3.60	6 months	①②③④
		China Modern Medicine	36	34	35.49 ± 3.59	35.21 ± 3.54	30.00 ± 4.00	30.00 ± 2.50	3 months	①②③④
		Jiangxi Medical Journal	38	38	34.22 ± 3.11	33.87 ± 3.21	30.50 ± 2.60	29.70 ± 2.80	3 months	②③④
		China & Foreign Medical Treatment	70	70	33.10 ± 4.20	33.00 ± 4.10	NR	NR	6 months	①②④
		Chin. J. of Clinical Rational Drug Use	100	100	33.30 ± 2.80	33.10 ± 2.70	18.00 ± 7.00	17.00 ± 6.00	6 months	①②④⑤
		Healthmust-Readmagazine	31	31	NR	NR	NR	NR	3 months	②③④
		SH. J. TCM	30	30	31.66 ± 5.53	31.89 ± 5.62	6.52 ± 2.18	6.46 ± 2.42	3 months	①②③④
		China Continuing Medical Education	50	50	33.67 ± 2.21	33.25 ± 2.67	37.50 ± 2.70	37.20 ± 2.10	3 months	①②③

Treatment measures	Control measures	Publication	Number of included cases		Age(year, x̄±s)		Course of disease (months, x̄±s)		Duration	Outcome
			Trial group	Control group	Trial group	Control group	Trial group	Control group		
KT+HRT	HRT	China Practice Medical	36	36	34.80 ± 4.10	34.40 ± 4.00	19.00 ± 5.00	18.00 ± 5.00	3 months	①②③④⑤
		Journal of New Chinese Medicine	35	35	33.20 ± 2.80	33.70 ± 2.40	18.00 ± 6.00	20.00 ± 7.00	3 weeks	①②③④
		Journal of Medical Theory and Practice	34	33	NR	NR	NR	NR	3 months	①②③④
		Maternal and Child Health Care of China	75	75	34.50 ± 3.70	34.10 ± 3.50	31.70 ± 9.20	32.50 ± 9.80	6 months	①②③④
		China Health Care and Nutrition	41	41	33.20 ± 1.40	33.80 ± 1.90	36.50 ± 3.60	37.20 ± 4.60	3 months	①②③④
		New Chinese Medicine	39	39	33.00 ± 1.40	33.00 ± 1.40	30.00 ± 4.00	30.00 ± 2.50	3 months	①②③④

P.S: NR, Not reported

Outcome: ①Total clinical response rate; ②FSH; ③LH; ④E2; ⑤Adverse reactions

HRT, hormone replacement therapy; YR, Fuke Yangrong Capsule; LWDH, Liuwei Dihuang Pills; XFZY, Xuefu Zhuyu Capsule; PK, Peikun pills; HCDZ, Heche Dazao pills; ZHC, Ziheche Capsule; SW, Siwu Mixture; ZG, Zuogui pills; GS, Guishen pills; ZSYT, Zishen Yutai pills; HS, Huanshao Capsule; KT, Kuntai Capsule.
